# The Apportionment of Pharmacogenomic Variation: Race, Ethnicity, and Adverse Drug Reactions

**DOI:** 10.18103/mra.v10i9.2986

**Published:** 2022-09-20

**Authors:** I. King Jordan, Shivam Sharma, Shashwat Deepali Nagar, Leonardo Mariño-Ramírez

**Affiliations:** 1 School of Biological Sciences, Georgia Institute of Technology, Atlanta, Georgia, USA; 2 IHRC-Georgia Tech Applied Bioinformatics Laboratory, Atlanta, Georgia, USA; 3 PanAmerican Bioinformatics Institute, Valle del Cauca, Cali, Colombia; 4 National Institute on Minority Health and Health Disparities, National Institutes of Health, Bethesda, Maryland, USA

## Abstract

Fifty years ago, Richard Lewontin found that the vast majority of human genetic variation falls within (~85%) rather than between (~15%) racial groups. This result has been replicated numerous times since and is widely taken to support the notion that genetic differences between racial groups are trivial and thus irrelevant for clinical decision-making. The aim of this study was to consider how the apportionment of pharmacogenomic variation within and between racial and ethnic groups relates to risk disparities for adverse drug reactions. We confirmed that the majority of pharmacogenomic variation falls within (97.3%) rather than between (2.78%) the three largest racial and ethnic groups in the United States: Black, Hispanic, and White. Nevertheless, pharmacogenomic variants showing far greater within than between-group variation can have high predictive value for adverse drug reactions, particularly for minority racial and ethnic groups. We predicted excess adverse drug reactions for minority Black and Hispanic groups, compared to the majority White group, and considered these results in light of the apportionment of genetic variation within and between groups. For 85% within and 15% between group variation, there are 700 excess adverse drug reactions per 1,000 patients predicted for a recessive effect model and 300 for a dominant model. We found high numbers of predicted Black and Hispanic excess adverse drug reactions for widely prescribed platinum chemotherapy compounds, such as cisplatin and oxaliplatin, as well as controlled narcotics, including fentanyl and tramadol. Our results indicate that race and ethnicity, while imprecise proxies for genetic diversity, correlate with patterns of pharmacogenomic variation in a way that is clearly relevant to medical treatment decisions. The effects of this variation is particularly pronounced for Black and Hispanic minority groups, owing to genetic differences from the majority White group. Treatment decisions that are made based on (assumed) White pharmacogenomic variant frequencies can be harmful for minority groups. Ignoring clinically relevant genetic differences among racial and ethnic groups, however well-intentioned, will exacerbate rather than ameliorate health disparities.

## Introduction

The question of whether race and ethnicity can serve as proxies for biological differences with relevance for clinical decision-making has received much attention as of late. Advocates for the continued use of race and ethnicity emphasize the importance of these categories for biological, social, and environmental determinants of health^1–3^. Critics hold that race and ethnicity are socially defined, rather than biological or anthropological categories, and emphasize the potential stigmatizing effects of race-based medicine^4–7^. There is, however, broad agreement that the use of race and ethnicity as markers of biological difference in the clinical setting can only be justified if the benefits outweigh the harm.

The extent of genetic differences that exist among racial and ethnic groups is central to this debate. Richard Lewontin first studied the apportionment of genetic variation among racial groups fifty years ago. His seminal paper showed that ~85% of human genetic variation was found within racial groups, compared to only ~15% between groups, and he concluded that race is “of no social value … is positively destructive of social and human relations … [and is] of virtually no genetic or taxonomic significance”^8^. Lewontin’s fundamental result has been confirmed numerous times since and is widely taken to support the irrelevance of genetic differences between racial and ethnic groups^9,10^. Here, we consider differences in the frequencies of pharmacogenomic variants^11^, which affect how individuals respond to medication and are thus particularly relevant to clinical decision-making, among the three largest racial and ethnic groups in the United States: Black, Hispanic, and White^12^. The aims of this study were (1) to characterize the apportionment of pharmacogenomic variation within and between groups and (2) to relate the apportionment of variation to disparities in the risk of adverse drug reactions (ADRs). There are more than two million serious ADRs among hospitalized patients in the US every year, leading to more than 100,000 deaths^13^. ADRs are a major public health problem and one that is largely preventable^14^; moreover, racial and ethnic minorities in the US bear a disproportionate burden of ADRs^15,16^. There are numerous pharmacogenomic variants associated with drug toxicity, which can be used to predict and avoid ADRs^17^.

Given what is known about the apportionment of human genetic variation, the majority of pharmacogenomic variation is expected to fall within rather than between racial and ethnic groups. If this proves to be the case, then race and ethnicity are expected to hold low predictive value for the genetic risk of ADRs^18^. In other words, according to the logic of Lewontin and his intellectual heirs, race and ethnicity are poor proxies for genetic diversity and thus irrelevant for pharmacogenomic informed therapeutic decision making. We tested these expectations via analysis of pharmacogenomic variation for a cohort of US study participants who self-identified as Black, Hispanic, or White, and via simulation of allele frequencies within and between groups for pharmacogenomic variants that are associated with drug toxicity.

## Materials and Methods

### Study cohort

Study participants were taken from the Michigan Health and Retirement Study (HRS), a national representative longitudinal panel study of Americans over the age of 50^19^. HRS participants self-identified their race and their ethnicity (SIRE) according to US Office of Management and Budget (OMB) standards^20^ and provided DNA samples for genome-wide genotype analysis. HRS participant genome-wide genotypes were characterized using the Illumina Omni2.5 BeadChip. Participant genotypes were filtered to remove variants with > 1% missingness and < 1% minor allele frequency among samples using PLINK v2^21^. The final genome-wide genotype dataset consists of 2,252,836 biallelic genetic variants.

### Apportionment of genetic variation

Genomic relationships among study participants were inferred via principal components analysis (PCA) of the participant genome-wide genotype data using the FastPCA program implemented in PLINK v2^21^.

The fixation index (*F*_*ST*_) was used to partition human genetic variation within (1−*F*_*ST*_) and between (*F*_*ST*_) SIRE group pairs^22^. *F*_*ST*_ values were calculated for individual genetic variants as follows:

1. The mean expected heterozygosity within each SIRE group $\left(\bar{H_{S}}\right)$ is calculated as the weighted average of variant heterozygosity within each group:
$$\bar{H_{S}}=\sum_{i}2\left(p_{i}\right)\left(1-p_{i}\right)\times \frac{\;count_{i}}{\;total\;count\;}$$
  where *p*_*i*_ is the frequency of the variant effect allele in group *i*, *count*_*i*_ is the number of individuals in group *i*, and *total count* is the sum of individuals in both groups.
2. The expected heterozygosity for the pair of both SIRE groups is calculated as:
$$H_{T}=2(\bar{p})(1-\bar{p})$$
  where $\bar{p}$ is the mean effect allele frequency for the variant across the pair of SIRE groups.
3. The fixation index (*F*_*ST*_) is calculated by combining expected variant heterozygosity values within groups $\left(\bar{H_{S}}\right)$ and for the pair of both groups (*H*_*T*_):
$$F_{ST}=1-\frac{\bar{H_{S}}}{H_{T}}$$

### Excess predicted adverse drug reactions

The numbers of excess predicted adverse drug reactions for Black and Hispanic minority groups, compared to the White majority reference group, were calculated for (1) simulated group-specific allele frequencies and (2) HRS group-specific allele frequencies for known pharmacogenomic variants. SIRE group-specific allele frequencies were considered together with SIRE group population fractions, taken from 2021 US Census data^23^, in order to apportion genetic variation within and between groups using the *F*_*ST*_ formulas shown in the previous section. SIRE group-specific allele frequencies were also used to calculate the excess number of predicted adverse drug reactions for recessive (two toxicity associated effect alleles needed) and dominant (one toxicity associated effect allele needed) modes of action.

1. Recessive model $\left(\hat{R_{ADR}}\right)$ of excess predicted adverse drug reactions:
$$\hat{R_{ADR}}=\left(p_{min}^{2}-p_{maj}^{2}\right)*1,000$$
  where $p_{min}^{2}$ is the homozygous genotype fraction for the minority SIRE group toxicity associated allele *p*, and $p_{maj}^{2}$ is the homozyogous genotype fraction for the majority SIRE group toxicity associated allele *p*.
2. Dominant model $\left(\hat{D_{ADR}}\right)$ of excess predicted adverse drug reactions:
$$\begin{array}{l} \hat{D_{ADR}}=\left(p_{min}^{2}-p_{maj}^{2}\right)*1,000\\ \;+(2*p_{min}*\left(1-p_{min}\right)-2\\ \;*p_{maj}*\left(1-p_{maj}\right))*1,000 \end{array}$$
  where 2 ∗ *p*_*min*_ ∗ (1 − *p*_*min*_) is the heterozygous genotype fraction for the minority SIRE group toxicity associated allele *p*, and 2 ∗ *p*_*maj*_ ∗ (1 − *p*_*maj*_) is the heterozygous genotype fraction for the majority SIRE group toxicity associated allele *p*.

### Pharmacogenomic variants

Pharmacogenomic variants with empirically supported toxic drug reaction associations were taken from the Pharmacogenomic Knowledge Database (PharmGKB), which provides manually curated pharmacogenomic variant annotations along with details on their drug response phenotypes^17^. Pharmacogenomic variant chromosomal locations, clinical annotations, effect allele identity, mode of effect (recessive or dominant), and evidence levels were taken from PharmGKB. Evidence levels correspond to pharmacogenomic variant-drug response association confidence: 1) high, 2) moderate, 3) low, 4) unsupported.

## Results

### Race, ethnicity, and genomic variation

We characterized the relationship between race, ethnicity, and genomic variation using a cohort of 8,912 participants from the University of Michigan Health and Retirement Study (HRS). The three demographic categories studied here correspond to participants who self-identified their race and ethnicity (SIRE) as non-Hispanic Black (1,527; 17.1%), Hispanic of any race (1,174; 13.2%), and non-Hispanic White (5,927; 66.5%). Group percentages correspond roughly to current US Census estimates, with a slight overrepresentation of Black (13.6% expected) and White (59.3% expected) participants compared to an underrepresentation of Hispanic (18.9% expected) participants. Additional non-Hispanic racial categories – American Indian or Alaska Native, Asian, Native Hawaiian or Other Pacific Islander – did not yield sufficient numbers of participants (n=284; 3.2%) for stratified analysis.

**Figure 1.**
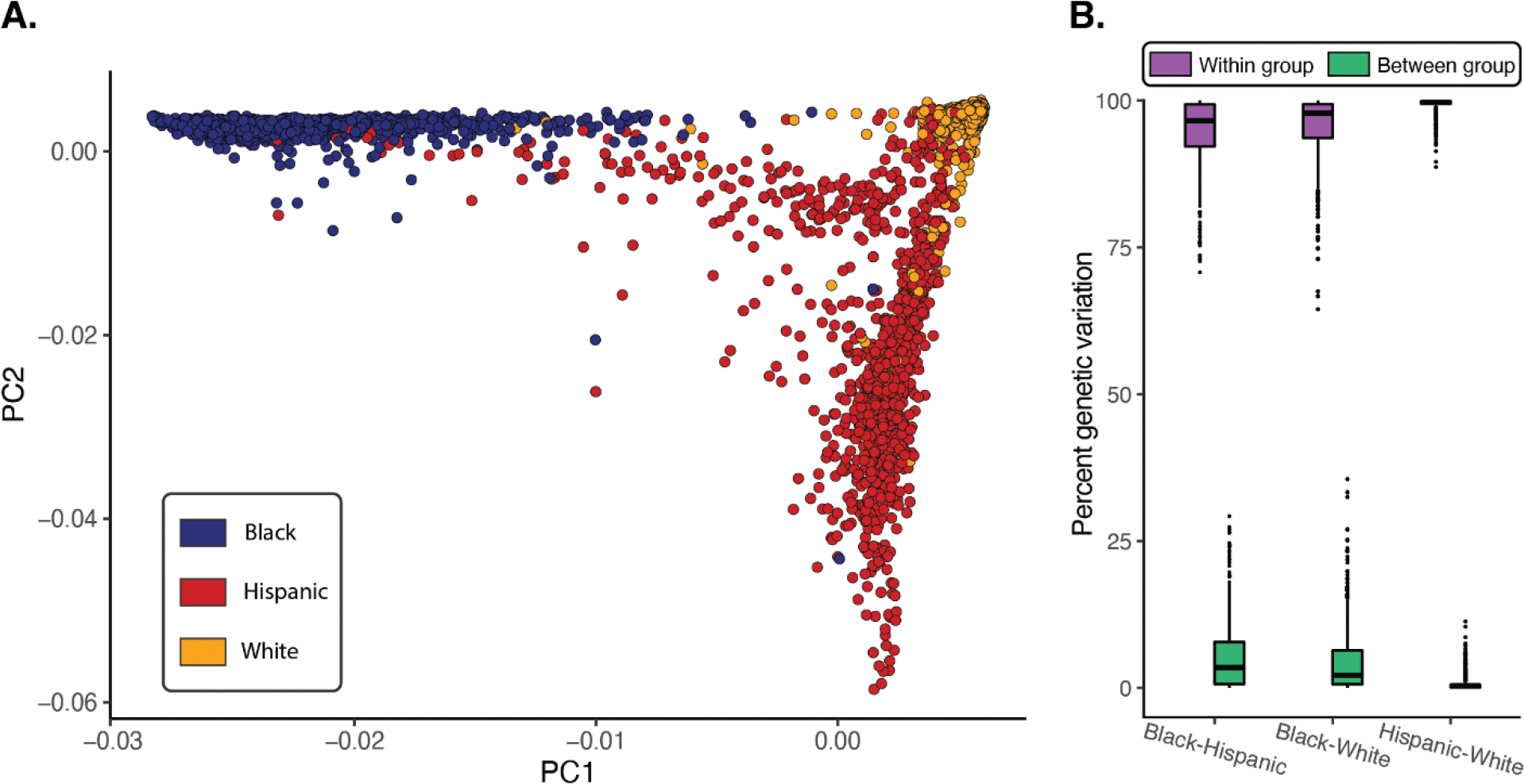
Race, ethnicity, and genomic variation. (A) Genomic relationships among study participants compared to their SIRE: Black (blue), Hispanic (red), and White (orange). (B) Apportionment of genetic variation within (purple) and between (green) SIRE group pairs.

The genomic relationships among study participants were characterized using principal components analysis (PCA) of genome-wide genotype data and visualized in light of participants SIRE (Figure 1A). The first two principal components (PC1 and PC2) capture 88% of the total genomic variation in the cohort, with clear differences and group-specific patterns of genomic variation corresponding to participants’ SIRE. The PCA plot shows three distinct poles of human genomic diversity: African ancestry (Figure 1A, upper left), European ancestry (Figure 1A, upper right), and Native American ancestry (Figure 1A, lower right), and participants from each SIRE group tend to segregate towards each corresponding pole. Nevertheless, there are no discrete boundaries between groups, and participants exhibit a continuum of genomic variation and admixture within and between groups. Hispanic participants in particular show a broad pattern genomic diversity, overlapping with both Black and White groups, consistent with the demographic definition of this group (individuals who self-identify Hispanic ethnicity can be of any race). Hispanic participants primarily show a continuum of admixture between European and Native American ancestry poles, an additional axis of admixture between European and African ancestry poles, and a number of participants with apparent three-way patterns of admixture. Black participants show a continuum of diversity and admixture from the African to the European ancestry pole, with a few individuals showing either three-way admixture or European-Native American admixture. White participants show the most coherent patterns of genomic diversity, and the least amount of apparent admixture, clustering tightly around the European ancestry pole.

We next compared the overall genomic diversity captured by PCA to the percent of genetic variation apportioned within and between pairs of SIRE groups, as measured the fixation index (F_ST_; Figure 1B). F_ST_ scales from 0–1 and measures the amount of genetic variation apportioned between population groups; 1− F_ST_ is taken as the amount of within group variation. F_ST_ values are calculated as shown in the Methods section, considering both SIRE group-specific allele frequencies and group population numbers. The vast majority of human genomic diversity is found within rather than between groups for all three pairwise SIRE group comparisons: Black-Hispanic average within group percent genetic variation=94.8% and between=5.2%, Black-White average within=96.3% and between=3.7%, Hispanic-White average within=99.5% between=0.5%. These results are consistent with the average values of 85% within and 15% between group variation found by Lewontin in 1972, with even more variation found within rather than between SIRE groups for the HRS participants.

**Figure 2.**
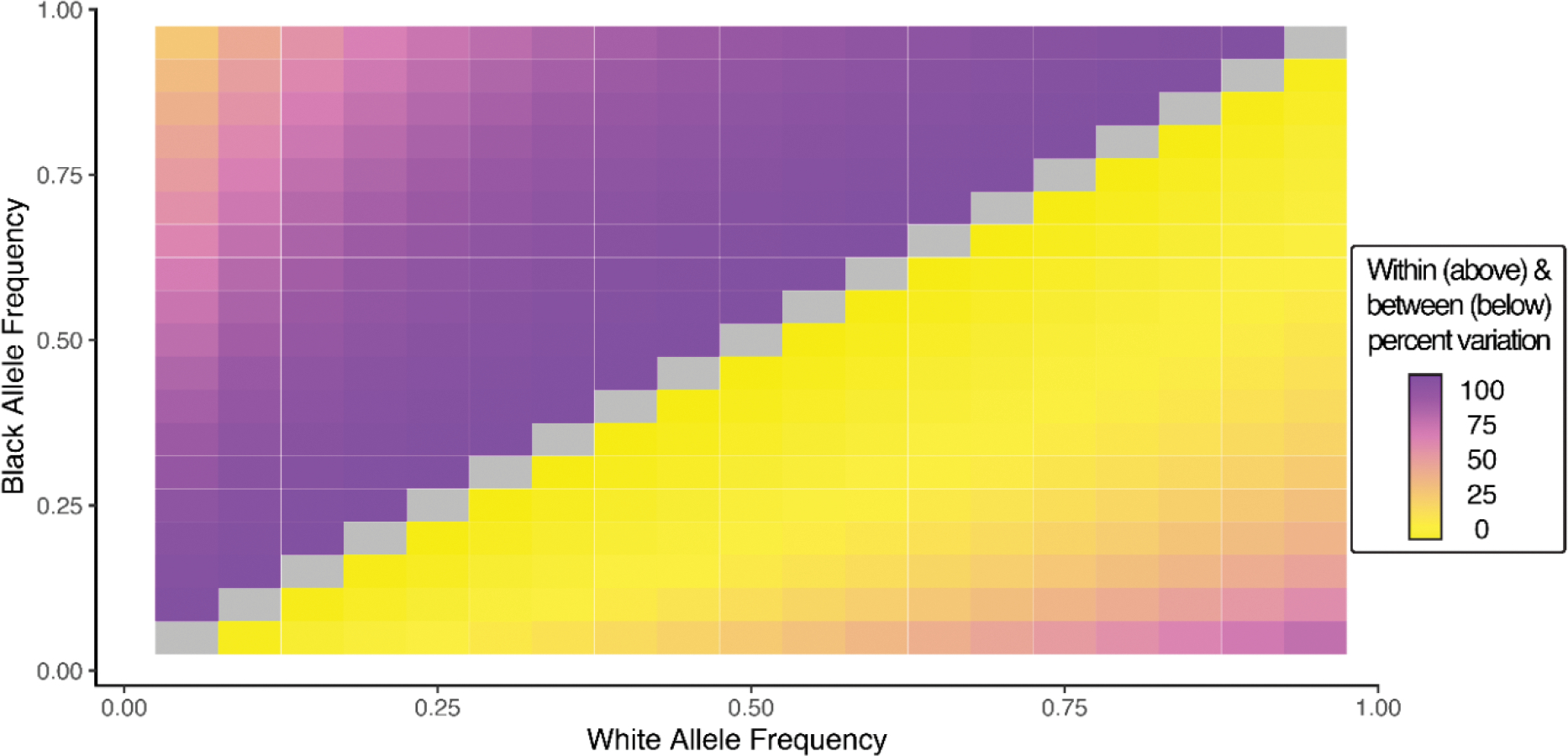
SIRE group allele frequencies and the apportionment of genetic variation. Pairwise comparison of Black and White group allele frequencies and the amount of genetic variation found within (above diagonal) and between (below diagonal) groups.

Finally, we simulated allele frequency divergence between SIRE group pairs to visualize the relationship between allele frequency divergence and the apportionment of genetic variation within and between groups. SIRE group-specific allele frequencies were simulated from 0–1, and the within (1− *F*_*ST*_) and between (*F*_*ST*_) group percent variation values were calculated for all possible pairs of group-specific allele frequencies. Results of this simulation are shown for comparisons of Black and White SIRE groups (Figure 2) and for Hispanic and White groups (Supplementary Figure 1). Relatively high levels of within group genetic variation can be seen across a very broad range of group-specific allele frequency values, whereas high between group variation is limited to a small range of extreme allele-frequency differences between groups. In other words, high levels of within versus between group genetic variation can persist in the face of substantial allele frequency divergence between groups.

### Apportionment of pharmacogenomic variation and adverse drug reactions

Simulation of SIRE group allele frequency divergence was also used to relate the apportionment of pharmacogenomic variation within and between groups to adverse drug reactions. There are numerous pharmacogenomic variants that are associated with adverse drug reactions caused by medication toxicity. SIRE group differences in allele frequencies for pharmacogenomic variants of this kind will lead to differences in toxic drug reactions between groups. We modeled the effect of SIRE group pharmacogenomic allele frequency differences on toxicity by measuring the predicted numbers of excess adverse drug reactions per 1,000 patients, taking the majority White study participants as the reference group compared to Black and Hispanic groups. This approach captures what would happen if majority White group allele frequencies of toxicity-associated pharmacogenomic variants are assumed to hold for minority Black and Hispanic groups. In other words, this approach simulates what would be expected to happen if patient race and ethnicity were not considered in treatment decisions of minority patients and thereby underscores the clinical implications of the apportionment of genetic variation within and between SIRE groups.

**Figure 3.**
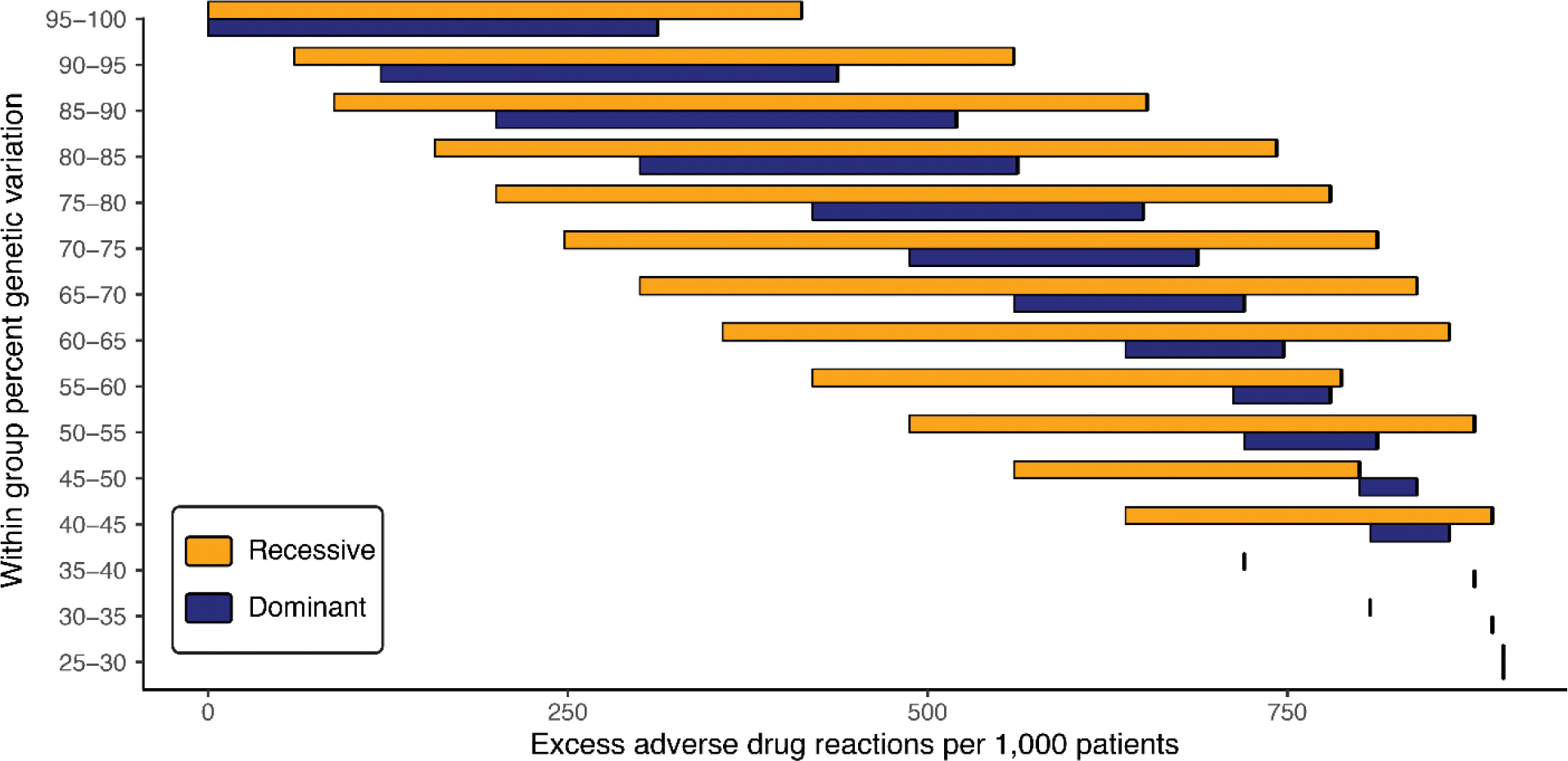
Apportionment of genetic variation and adverse drug reactions. Results are shown for a comparison of Black (minority) and White (majority) SIRE groups. The amount of within group genetic variation (y-axis) is compared to ranges of the predicted number of excess adverse drug reactions (x-axis) for the minority Black group. Results are shown for recessive (yellow) and blue (dominant) pharmacogenomic variant effect modes.

There are large numbers of predicted excess adverse drug reactions for minority Black (Figure 3) and Hispanic (Supplementary Figure 2) groups, compared to the majority White group, across almost all values of within versus between group genetic variation. This holds for both recessive and dominant effect modes of pharmacogenomic variation-drug toxicity associations. For the Black-White group comparison, at the highest levels of within group variation (95–100%), the numbers of predicted excess adverse drug reactions range from 0–412 for the recessive model and 0–312 for the dominant model. For 85% within and 15% between group variation, the apportionment of human genetic variation found by Lewontin, there are 700 predicted excess adverse drug reactions for the recessive model and 300 for the dominant model. At equal levels of within and between group variation, at least half or more of all minority patients are predicted to have adverse drug reactions, irrespective of recessive or dominant mode of action. Similar results can be seen the Hispanic-White comparison, and results for all comparisons are shown in Supplementary Table 1. We mined the PharmGKB database to evaluate the relationship between the apportionment of genetic variation within and between SIRE groups and empirically validated associations between pharmacogenomic variants and adverse drug reactions. We uncovered 1,075 drug toxicity pharmacogenomic variant associations across all four levels of evidence. The vast majority of genetic variation for these variants falls within rather than between pairs of SIRE groups studied here: Black-Hispanic (within=96.8%, between=3.2%), Black-White (within=95.7%, between=4.3%), Hispanic-White (within=99.4%, between=0.6%). Nevertheless, there are numerous pharmacogenomic variants with allele frequency differences between groups that are predicted to yield large numbers of excess adverse drug reactions in minority racial and ethnic groups. Table 1 shows the top 5 variants each, as measured by the numbers of predicted excess adverse drug reactions for Black and Hispanic minority groups, compared to the White majority group.

The toxicity-associated pharmacogenomic variant rs11615 is a synonymous A/G variant in the *ERCC1* (Excision Repair 1, Endonuclease Non-Catalytic Subunit) protein coding gene. The G effect allele is found at high frequency in Black (0.88) and Hispanic (0.64) study participants compared to White (0.37) participants, leading to predicted excesses of 629 (recessive) and 379 (dominant) adverse drug reactions per 1,000 Black patients and predicted excesses of 629 (recessive) and 379 (dominant) adverse drug reactions per 1,000 Hispanic patients, compared to the White majority group. This variant is associated with adverse reactions to 9 different drugs, including the widely prescribed cisplatin and oxaliplatin chemotherapy drugs. Several other variants with large numbers of predicted excess adverse reactions are associated with platinum chemotherapy compounds as well as other widely prescribed drugs, such as the controlled narcotics fentanyl, haloperidol, and tramadol. A full list of predicted excess adverse drug reactions for toxicity associated variants can be found in Supplementary Table 2.

## Discussion

Our analysis of the HRS participant cohort illuminates the relationship between race, ethnicity, and genetics for the three largest racial and ethnic groups in the US: Black, Hispanic, and White. While we do find obvious genetic differences between these three demographic groups, there are no discrete boundaries between groups. Rather, our cohort shows a continuum of genetic diversity and admixture, with the vast majority of variation falling within rather than between groups. Our results are consistent with previous large-scale studies of the US population, all of which show characteristic group-specific patterns of diversity coupled with a continuum of admixture within and between groups^24–26^.

Fifty years ago, in the pre-DNA sequencing era, Richard Lewontin marshalled data on protein polymorphisms to show that the vast majority of human genetic diversity falls within rather than between racial groups. This seminal result has held up remarkably well over the years, through the invention of DNA sequencing and well into the genomic era^9,10^. Lewontin concluded that race is both a destructive force in human relations and a construct that has virtually no genetic significance. His admonition has been widely adopted as an argument against any consideration of whatsoever of race and ethnicity in genetics research and clinical decision making^10^. We show here that clinically relevant genetic differences between racial and ethnic groups can exist even when there is far more variation within rather than between groups. In particular, if Black and Hispanic minority patients in the US are treated based on the assumption of no meaningful genetic differences with the majority White group, they are likely to suffer numerous, and largely avoidable, adverse drug reactions.

Our results point to platinum-based cancer therapeutics (platins) as an example of how patient race and ethnicity can be used to inform treatment decisions. Platins, such as cisplatin, oxaliplatin, and carboplatin, are used to treat almost half of cancer patients receiving chemotherapy^27^, and they are known to cause a wide array of adverse effects^28^. We found a number of platin-associated pharmacogenomic variants with toxicity risk alleles at elevated frequencies in minority Black and Hispanic populations (Table 1 and Supplementary Table 2). For platins that are counter-indicated by patients’ race or ethnicity, such as the case of the *ERCC1* variant rs11615, there are several options that can be used to mitigate the risk of toxicity. First and foremost, alternative medications can be prescribed. For example, since the rs11615 effect allele is counter-indicated for cisplatin and oxaliplatin, the alternative carboplatin could be prescribed for at risk patients^29^. In cases where alternative platin treatments are not available, premedication, e.g. with antihistamines or corticosteroids, skin testing, and desensitization protocols have all been shown to mitigate hypersensitivity reactions to platins^30^. To be clear, we are not advocating that race and ethnicity alone be used to make treatment decisions of this kind, but they can serve as a valuable source of information at physicians’ disposal.

The use of race and ethnicity as a proxy for genetic differences with clinical relevance can only be justified if (1) benefits outweigh harm and (2) there are no alternative methods for patient stratification with equal or superior accuracy. Objections to the use of race and ethnicity in genetics research and clinical decision making are grounded in entirely reasonable concerns about the potential stigmatizing effects of highlighting biological and genetic differences between racial and ethnic groups^31–33^. Indeed, race and ethnicity are widely recognized as social rather than biological or genetic categories^34–38^. Nevertheless, ignoring race and ethnicity in treatment decisions has the potential to cause tangible harm to patients. The harm to patients caused by the race-blind approach is exemplified by the case of the blood thinner Plavix in Hawaii^39^. In 2021, the pharmaceutical companies Bristol-Myers Squibb and Sanofi were ordered to pay $834 million to the state based on their failure to disclose the drug’s diminished effects in Asian patients compared to Black or White patients. In 2015, adverse reactions to the government prescribed HIV medication efavirenz occurred among ~20% of patients in Zimbabwe, despite previous warnings of a genetic variant found at high frequency in the population that is associated with slow metabolism and accumulation of the drug in the bloodstream^40,41^. As we have shown here, there are scores of drugs that present similar, and perhaps even more extreme, dangers of adverse drug reactions to Black and Hispanic patients in the US.

The social definition of race and ethnicity is supported by the fact that racial and ethnic group delineations change over time and space. In the US, racial and ethnic classification have changed 20 times since the first US Census in 1790, which contained only three race categories: White, All Other Free Persons, and Slaves^42^. The Hispanic ethnic category was added in 1980, and race and ethnicity are now treated separately in the US census. Other cosmopolitan countries have their own systems of racial and ethnic classification, which may or may not be similar to that of US. The United Kingdom uses the term ethnicity in a way that is analogous to race in the US, but their ethnic categories can differ from the racial categories in the US owing to the pattern on immigration from Commonwealth countries. For example, the Asian census category in the UK refers primarily to individuals of Indian, Pakistani, and Bangladeshi origin. At the global level, genetic diversity is largely continuously distributed, based on reproductive isolation by distance, with discontinuities introduced by major geographic barriers (e.g. mountains, deserts, and oceans) and social barriers (e.g. assortative mating). Accordingly, the extent to which race and ethnicity can serve as proxies for genetic diversity will depend on historical patterns of immigration and current demography for any given country. The three largest racial and ethnic groups in the US – Black, Hispanic, and White – include individuals with ancestry from geographically diverse regions – Africa, the Americas, and Europe – home to populations that evolved separately for many thousands of years before coming together in the post-Columbian era. Thus, US racial and ethnic groups can be readily genetically distinguished, since genetic diversity closely tracks continental ancestry^43,44^. This fact is underscored by a recent study of >200,000 US military veterans, which showed 99.47% correspondence between genome-wide patterns of genetic diversity and participant SIRE^25^. Nevertheless, as the US and other cosmopolitan nations become increasingly diverse, owing to ongoing immigration and increasing rates of intermarriage, the correspondence between race, ethnicity, and genetic diversity is expected to break down.

Finally, it is worth reiterating that race and ethnicity remain imprecise proxies for genetic diversity, particularly for individual genetic loci, and it must be stressed that there are alternative methods that provide greater accuracy for patient stratification. Genetic ancestry is a far more precise proxy for genetic diversity, and a superior means for stratifying patient populations^1,6^, and pharmacogenomic genotyping is an even more direct way to assess the presence of toxicity associated variants^45^. If genomic and genetic technologies of these kinds were widely available for patients, then race and ethnicity would indeed be rendered irrelevant for therapeutic decision making. However, minority populations continue to be vastly underrepresented in clinical genetics cohorts^46–49^ and are less likely to have access to genomic medicine technologies^50,51^. Until these disparities in research and health care access are rectified, race and ethnicity should continue to serve as a tool for pharmacogenomic patient stratification.

## Conclusions

When it comes to life and death clinical treatment decisions, race and ethnicity clearly matter. Our results demonstrate that the therapeutic relevance of patient race and ethnicity hold despite the demonstrably true facts that (1) race and ethnicity are imprecise proxies for genetic diversity and (2) the vast majority of human genetic variation falls within rather than between demographic groups. Genetic differences between patients that self-identify as belonging to different racial and ethnic groups are nonetheless highly predictive of adverse drug reactions, and this is especially true for minority populations with genetic profiles that differ from the majority group. Disregarding patient race and ethnicity, however well intentioned, will exacerbate rather than alleviate health disparities.

## Supplementary Material

Supplementary Table 1

Supplementary Table 2

Supplementary Figures 1 and 2

## Figures and Tables

**Table 1.** Apportionment of pharmacogenomic variation and excess adverse drug reactions. Pharmacogenomic variants with high numbers of predicted excess adverse drug reactions are shown for Black-White and Hispanic-White SIRE group comparisons.

**Black – White Excess Adverse Drug Reactions (ADRs)**								
**Variant** ^1^	**Gene** ^2^	**Drug(s)** ^2^	**f(Black)** ^3^	**f(White)** ^3^	**% Witdin** ^4^	**% Between** ^4^	**Excess ADRs** ^5^	
							**Recessive**	**Dominant**
chr19:45923653 (rsl 1615)	ERCC1	Cisplatin, fluorouracil, leucovorin, oxaliplatin, doxorubicin, cyclophosphamide, docetaxel, bleomycin, etoposide	0.88	0.37	0.83	0.17	629	379
chr7:154072020 (rs6977820)	DPP6	Antipsychotics	0.74	0.27	0.84	0.16	481	464
chr13:28624294 (rs1 933437)	FLT3	Sunitinib	0.66	0.40	0.95	0.05	279	248
chr1:1 1 856378 (rs1 801 1 33)	CLCN6, MTHFR	Methotrexate	0.90	0.66	0.96	0.04	364	103
chr22:1 9951271 (rs4680)	COMT	Fentanyl, buprenorphine, tramadol, haloperidol, nicotine	0.70	0.48	0.97	0.03	252	176
**Hispanic – White Excess Adverse Drug Reactions (ADRs)**								
**Variant** ^1^	**Gene** ^2^	**Drug(s**)^2^	**f(Hispanic**)^3^	**f(White**)^3^	**% Within** ^4^	**% Between** ^4^	**Excess ADRs** ^5^	
							**Recessive**	**Dominant**
chr19:45923653 (rs1 1615)	ERCC1	Cisplatin, fluorouracil, leucovorin, oxaliplatin, doxorubicin, cyclophosphamide, docetaxel, bleomycin, etoposide	0.64	0.37	0.96	0.04	275	267
chr19:4591 2736 (rs3212986)	CD3EAP, ERCC1, PPP1R13L	Paclitaxel, bleomycin, cisplatin, etoposide, docetaxel, cyclophosphamide, doxorubicin, fluorouracil	0.35	0.25	0.993	0.007	60	141
chr22:1 9951271 (rs4680)	COMT	Buprenorphine, fentanyl, tramadol, haloperidol	0.58	0.48	0.995	0.005	104	92
chr1:1 1 856378 (rs1 801 133)	CLCN6, MTHFR	Methotrexate, cisplatin, oxaliplatin, Platinum compounds, doxorubicin, nitrous oxide, phenobarbital, phenytoin	0.42	0.33	0.996	0.004	65	106
chr6:154360797 (rs1799971)	OPRM1	Fentanyl, cannabinoids, morphine, methadone, buprenorphine, tramadol, alfentanil, hydrocodone, tianeptine, heroin	0.20	0.13	0.995	0.005	22	112

1 Pharmacogenomic variant chromosomal position and variant (SNP) identifier.

2 Variant associated gene and drugs with documented toxic effects. Results are limited to PharmGKB evidence levels 1 –2.

3 Pharmacogenomic effect allele frequencies for each SIRE group.

4 Percent of variation within and between SIRE groups.

5 Predicted excess adverse drug reactions for the minority (Black or Hispanic) group compared to the majority White group.
