# Supplementary material for: The Apportionment of Pharmacogenomic Variation: Race, Ethnicity, and Adverse Drug Reactions
Source: Med Res Arch. Author manuscript; Available in PMC 2022 Oct 26. (PMC9600569; doi:10.18103/mra.v10i9.2986)
Supplement: Supplementary Figures 1 and 2 [file NIHMS1841078-supplement-Supplementary_Figures_1_and_2.docx]

**Supplementary Information**

**The apportionment of pharmacogenomic variation: race, ethnicity, and adverse drug reactions**

I. King Jordan, Shivam Sharma, Shashwat Deepali Nagar, and Leonardo Mariño-Ramírez


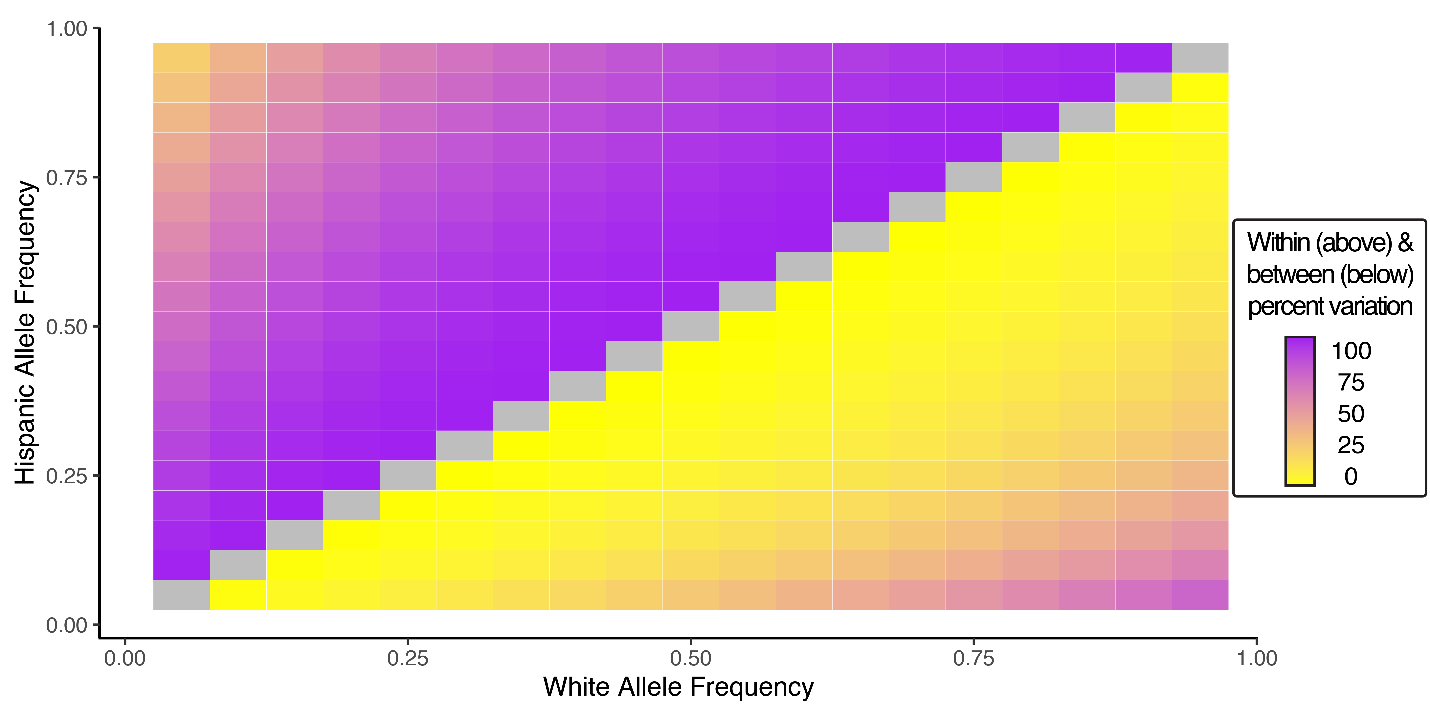


Supplementary Figure 1. **SIRE group allele frequencies and the apportionment of genetic variation.** Pairwise comparison of Hispanic and White group allele frequencies and the amount of genetic variation found within (above diagonal) and between (below diagonal) groups.


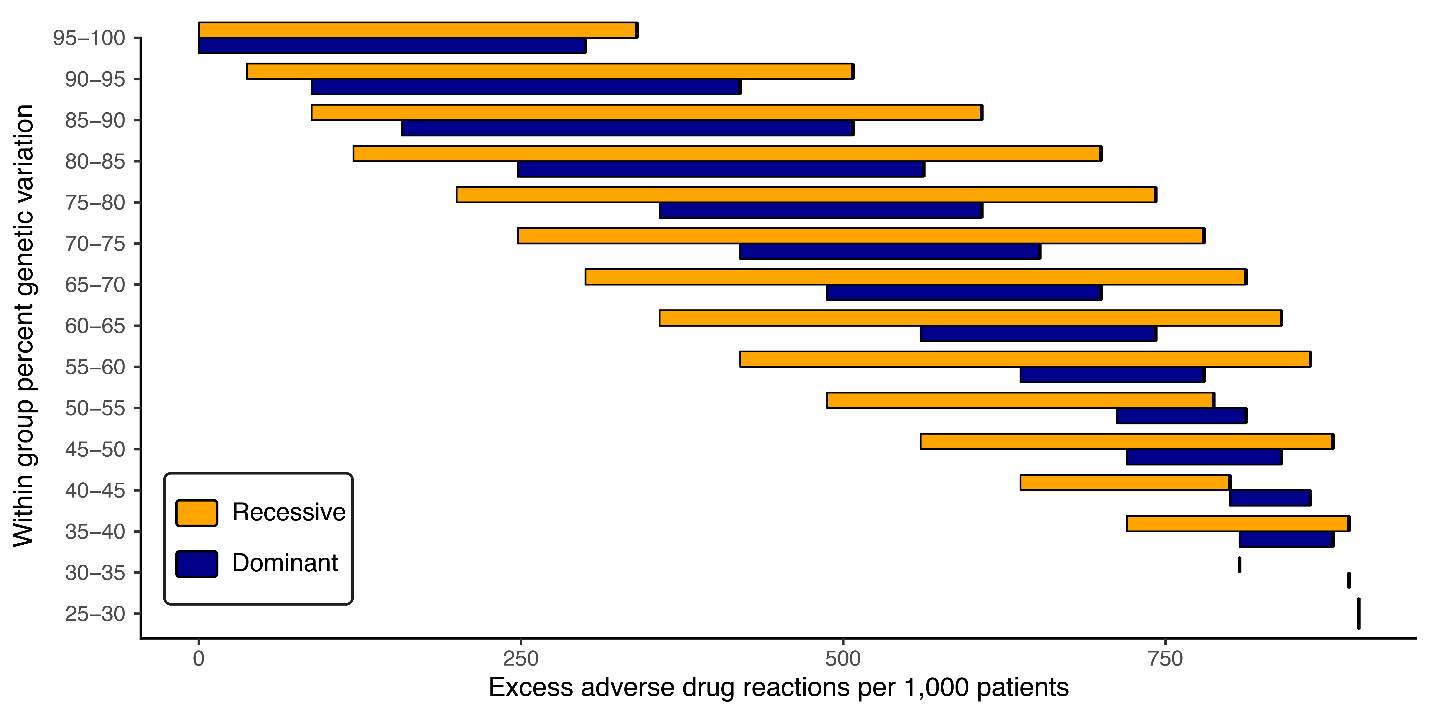


Supplementary Figure 2. **Apportionment of genetic variation and adverse drug reactions.** Results are shown for a comparison of Hispanic (minority) and White (majority) SIRE groups. The amount of within group genetic variation (y-axis) is compared to the predicted number of excess adverse drug reactions (x-axis) for the minority Hispanic group. Results are shown for recessive (yellow) and blue (dominant) pharmacogenomic variant effect modes.
